# Examining the Independent and Interactive Carryover Effects of Cognitive and Physical Exertions on Physical Performance

**DOI:** 10.1177/00187208241293720

**Published:** 2024-10-21

**Authors:** Rahul K. Pabla, Jeffrey D. Graham, Michael W. B. Watterworth, Nicholas J. La Delfa

**Affiliations:** 1 85458 Ontario Tech University, Canada

**Keywords:** ergonomics, mental fatigue, muscle fatigue, handgrip endurance, strength

## Abstract

**Objective:**

This study compared the effects of prior cognitive, physical, and concurrent exertion on physical performance.

**Background:**

Fatiguing cognitive and physical exertions have been shown to negatively affect subsequent task performance. However, it is not clearly understood if concurrent physical and cognitive effort may exaggerate the negative carryover effects on physical task performance when compared to cognitive or physical exertion alone.

**Method:**

Twenty-five participants completed four isometric handgrip endurance trials on different days. The endurance trials were preceded by four, 15-minute experimental manipulations (cognitive, physical, concurrent, control). Electromyography (EMG) and force tracing performance were monitored, with handgrip strength measured pre and post. Subjective ratings of mental and physical fatigue, as well as affect, motivation, and task self-efficacy, were also assessed.

**Results:**

Handgrip strength decreased following both physical (−14.4% MVC) and concurrent (−12.3% MVC) exertion manipulations, with no changes being observed for the cognitive and control conditions. No differences were observed across conditions for endurance time, EMG, nor tracing performance. When compared to the control conditions, perceptions of mental and physical fatigue were higher following the experimental manipulation. Endurance trial self-efficacy was lower for the mental, physical and concurrent conditions compared to control.

**Conclusion:**

The concurrent condition resulted in similar decreases in strength as the physical fatigue condition, but otherwise resulted in similar carryover effects on endurance performance across all conditions. Further study is required at higher exposure levels, or for longer exposure durations, to further probe the influence of concurrent physical and cognitive effort on task performance.

**Application:**

Concurrent cognitive and physical effort resulted in similar physical performance decrements to physical effort alone.

## Introduction

Individually, both muscle and cognitive fatigue can have negative carryover effects on various aspects of physical and/or cognitive task performance. Muscle fatigue results from prolonged and/or repetitive physical exertion and is often observed through reductions in muscular strength, speed, and endurance, as well as alterations in neurophysiological processes and increased perceptions of fatigue (Enoka & Duchateau, 2016; Gandevia, 2001; Mickelwright et al., 2017). Prolonged physical task performance can also reduce cognitive performance (e.g., Brown & Bray, 2015). Similarly, a growing body of literature has focused on how prolonged cognitive exertion, and subsequent perceptions of mental fatigue, can lead to reductions in both cognitive (Boksem & Tops, 2008) and physical task performance (Marcora et al., 2009), including strength, endurance, and motor control (Brown et al., 2020). Reductions in task performance often occur in tandem with increased perceptions of mental fatigue (Brown & Bray, 2017; Clarkson et al., 2016; Marcora et al., 2009). Taken together, there is accumulating evidence that both excessive physical and cognitive exertion can have negative carryover effects on both physical and cognitive task performance.

It is important to note that previous studies, which have examined the negative carryover effects of either physical or cognitive exertion on subsequent task performance, have primarily used a sequential task design; whereby participants initially perform a control or experimental task followed by the dependent measure of task performance. While the experimental designs of these studies may slightly differ (e.g., two vs. three or more sequential tasks and between- vs. within-subject designs), they ultimately seek to understand how an initial task that requires high levels of physical or cognitive exertion (when compared to a control condition) negatively alters performance on a subsequent task that primarily requires physical or cognitive resources. Moreover, within a two-task carryover paradigm, typical experimental designs can either include: (1) physical versus control manipulation and a physical dependent measure, (2) physical versus control manipulation and a cognitive dependent measure, (3) cognitive versus control manipulation and a cognitive dependent measure, and (4) cognitive versus control manipulation and a physical dependent measure. Of particular interest, these experimental designs are primarily focused on how cognitive or physical exertion alone (when compared with a control condition) may have negative carryover effects on cognitive or physical task performance. However, many tasks require varying levels of both physical and cognitive exertion, and this concurrent exertion may have exacerbated negative carryover effects when compared to cognitive or physical exertion alone, warranting further investigation. This is especially relevant as many occupational tasks require individuals to exert physical and cognitive effort at the same time on one task and then immediately perform another task (e.g., surgery, automotive repair, and electrical work). However, it is not clearly understood if concurrent fatiguing bouts of both physical and cognitive effort may exaggerate the negative carryover effects on task performance when compared to cognitive or physical exertion alone.

To date, only a handful of studies have examined the carryover effects of concurrent physical and cognitive exertion on physical and/or cognitive task performance. For instance, previous research found no differences between a concurrent exertion condition (physical and cognitive) when compared to a physical exertion alone condition on levels of muscular strength within the forearm (Mehta & Parasuraman, 2014; Yoon et al., 2009) and shoulder (Mehta & Agnew, 2012) muscles. However, these studies did not include a distinct cognitive exertion alone condition nor a control condition, which limits our ability to understand how different types of exertion may compare to one another in relation to indices of muscular strength. It would also be worthwhile to replicate these initial studies with a full factorial design (i.e., all experimental conditions) and investigate the potential negative carryover effects on physical endurance performance and motor control, given previous research demonstrating the negative carryover effects of physical exertion and cognitive exertion on physical performance previously discussed.

A relevant study by Coutinho et al. (2018) investigated the carryover effects of physical exertion alone, cognitive exertion alone, or a control condition on various aspects of soccer performance. Findings revealed that the cognitive exertion condition had the greatest negative effect on soccer performance when compared to the physical exertion alone and control conditions. In addition, the physical exertion condition had inconsistent carryover effects on soccer performance (some negative and some positive) when compared to the control condition. While this study builds on previous research (e.g., Mehta & Agnew, 2012), it is still limited as it did not include a concurrent exertion condition. Finally, a recent study by Alder et al. (2021) included all possible experimental conditions (i.e., control, physical alone, cognitive alone, and concurrent) within a single study and examined the carryover effects of the various types of exertion conditions on anticipatory decision-making performance using a soccer film test. When compared to the control condition, findings revealed that the concurrent condition had the greatest negative effect on performance, followed by the cognitive and physical exertion alone conditions. Performance in the cognitive and physical exertion conditions was not significantly different. This preliminary research suggests that concurrent cognitive and physical exertion may have exacerbated effects on performance when compared to physical or cognitive exertion alone.

While the examination of the potential negative carryover effects of concurrent cognitive and physical exertion is still emerging, insight as to why concurrent exertion may have greater negative effects can be drawn from previous research that include a combination of possible physiological and psychological mechanisms. For instance, the negative carryover effects of cognitive and physical exertion have been suggested to affect various neurophysiological pathways contributing to subsequent reductions in task performance and are linked to the concept of “whole-body fatigue” which can be observed within the muscles through reductions in strength and endurance (e.g., Gandevia, 2001; Martin et al., 2018; Noakes, 2012; Weavil & Amann, 2019). Taken together, physical and cognitive fatigue should have compounding negative effects on the human body’s neurophysiological processes and functioning. Other research has also theorized and shown that physical and cognitive exertion can have independent carryover effects on various psychological processes which, and importantly, contribute to subsequent task performance (e.g., Bandura, 1997; Martin et al., 2018). That is, prior physical and cognitive exertion has been suggested to negatively affect perceptions of self-efficacy and fatigue (Bandura, 1997; Graham et al., 2017; Moritz et al., 2000; Samson & Solmon, 2011), affect (Ekkekakis & Brand, 2019; Hagger et al., 2010), and motivation (Inzlicht et al., 2014; Martin et al., 2018; Molden et al., 2016; Teixeria et al., 2012). Collectively, alterations in physiological and psychological processes resulting from concurrent cognitive and physical exertion should have additive negative effects on performance when compared to individual physical or cognitive exertion. However, this has yet to be examined within a single study.

The lack of previous research examining comparisons between the independent and interactive effects of prior cognitive and physical exertion on task performance within a single study is somewhat surprising given the known independent and concurrent physical and cognitive demands within various occupations (e.g., surgery, dentistry, and assembly line tasks) as well as across various sport- and exercise-based tasks that require endurance, strength, accuracy, and decision making. As previously stated, while prior research has examined many combinations of comparisons (e.g., physical vs. cognitive, cognitive vs. concurrent, etc.) it is important to investigate each possible comparison within a single study to provide adequate data and advance our understanding of possible effects. For instance, Coutinho et al.’s study (2018) included three of the possible combinations on soccer performance except for a concurrent condition. Based on previous literature (e.g., Mehta & Parasuraman, 2014) it could be hypothesized that a concurrent condition would not be different from the physical exertion condition in the Coutinho et al. (2018) study, yet these studies used different physical task outcomes (soccer performance vs. handgrip strength) for which comparisons should not be made across studies. Therefore, there is impetus within the extant literature for studies to be completed that include all possible combinations of exertions (i.e., cognitive, physical, concurrent, and control conditions) to adequately provide complete data across various tasks. To date, only the Alder et al. (2021) study has included all possible conditions; however, their task outcome was cognitive in nature which highlights the need for research examining physical task outcomes.

The purpose of this study was to evaluate the independent (i.e., physical vs. cognitive) and interactive (i.e., physical AND cognitive) carryover effects of these exertions, relative to a control condition, on various physical outcome measures (e.g., strength, endurance, physical task performance, and electromyography). We hypothesized that concurrent cognitive and physical exertion would have the greatest negative carryover effect on physical task performance when compared to a control condition. We also hypothesized cognitive and physical exertion alone would each have negative carryover effects on all outcome variables, but these would not be as pronounced as the concurrent condition. Finally, we hypothesized that self-efficacy, affect, and motivation would be negatively affected by prior cognitive, physical, and concurrent exertion.

## Methods

### Experimental Design

The study utilized a within-subject repeated-measures design with four separate experimental conditions. The study consisted of five visits to the lab, and each visit was separated by at least three days to allow for adequate recovery between sessions. The first visit served as a familiarization session and to obtain baseline grip endurance performance. The presentation of experimental conditions on subsequent visits was counterbalanced to minimize any learning or order effects.

### Participants

25 university students (*n* = 12 females; *n* = 23 right-handed) were recruited into the study. Participants had a mean age of 21.6 ± 3.18, stature of 1.71 ± 0.11 m, mass of 70.4 ± 19.1 kg, and BMI of 24.0 ± 5.67 kg/m^2^. Exclusion criteria included shoulder and forearm injury or pain in the past 12 months, and any high-performance athletes (e.g., varsity athletes or similar level and above), whose performance may be affected by their skill level and/or tolerance to pain. This research complied with the tenets of the Declaration of Helsinki and was approved by the Institutional Review Board at Ontario Tech University. Informed consent was obtained from each participant.

### Data Acquisition and Instrumentation

#### Handgrip Dynamometer

**Figure 1.**
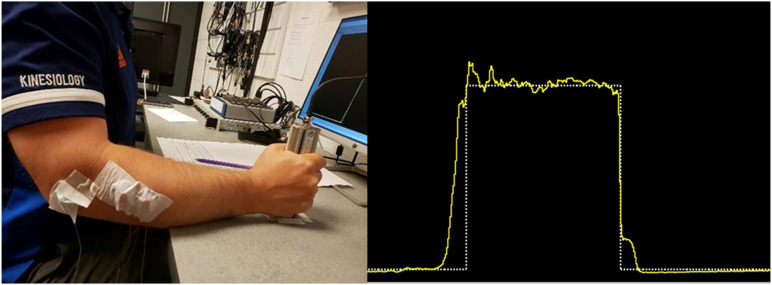
Left: Participant interaction with the handgrip dynamometer in the standardized experimental posture. Right: Example of the real-time force feedback provided to participants via custom LabView program. Grip forces were normalized to maximum grip strength.

A Lorenz Messtechnik K-2565 handgrip dynamometer was utilized to obtain grip forces. Signals were sampled at 2000 Hz using a 16-bit National Instruments analog to digital device. The force signal was smoothed using a half-second moving average with online visual force feedback provided on a monitor located in front of the participant using custom LabVIEW software. The handgrip dynamometer remained in a vertical orientation at a constant location on the table, promoting a neutral pronation/supination posture (Figure 1). A 90-degree elbow angle was maintained by adjusting a height-adjustable chair according to each participant’s anthropometry. As such, participants’ forearms were hovering a couple centimeters above the table’s surface to avoid any contact stress. The grip span of the device was not-adjustable; however, all workloads were normalized to each participant’s MVC at this grip span and test posture, allowing for consistent submaximal relative efforts during the experimental protocol.

#### Electromyography

Surface electromyography (sEMG) was used to record muscle activity for the flexor carpi radialis (FCR), flexor carpi ulnaris (FCU), and the extensor carpi radialis (ECR) of the dominant arm. Disposable bipolar Ag/AgCl surface electrodes with foam adhesive hydrogel (disc-shaped, 3 cm radius; Meditrace 130, Kendall, Mansfield, MA, USA) were placed on the skin overlying the muscle belly after shaving, abrading, and cleaning the area with an isopropyl alcohol swab. sEMG was detected and amplified using a Bortec AMT-8 system and was sampled at 2000 Hz with the National Instruments A/D card.

### Experimental Protocol

**Figure 2.**
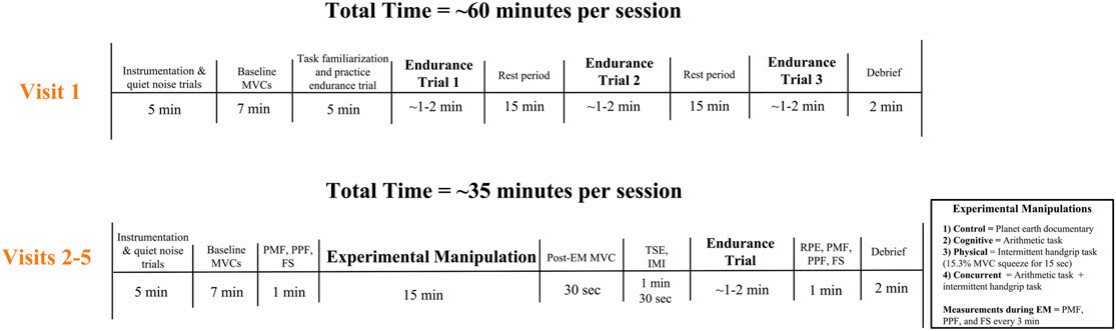
A flowchart of the experimental protocol. *Note.* Experimental protocol. EM = experimental manipulation, MVC = maximum voluntary contraction, min = minute, sec = seconds, PMF = perceived mental fatigue, PPF = perceived physical fatigue, FS = feeling scale, TSE = task self-efficacy, IMI = intrinsic motivation inventory effort and importance subscale, RPE = ratings of perceived exertion.

This study required participants to visit the lab on 5 separate days that were separated by at least 72 hours. See Figure 2 for an overview of the experimental protocol.

#### Familiarization (Visit 1)

During the familiarization session, participants were instrumented with sEMG. Next, a quiet noise trial was collected for both the sEMG and handgrip dynamometer for the purposes of de-biasing both signals. Following the noise trial, participants performed three baseline handgrip MVCs with 2 minutes of rest between each MVC. All grip strength MVCs were collected in a seated position with participants’ arms by their side, elbow flexed to 90° and wrist maintaining a neutral posture (Roberts et al., 2011). To complete an MVC, participants were asked to squeeze the handgrip dynamometer as hard as possible for 5 seconds. In the same arm posture as the grip MVCs, participants then performed one MVC for wrist extension and one MVC for wrist flexion, in case these contractions elicited a higher peak sEMG amplitude for signal normalization.

Participants then completed a 10-second practice endurance trial at 50% MVC to familiarize themselves with the task requirements and demands. Following a 3-minute rest period, participants completed three endurance trials to exhaustion, with 15 minutes of rest between each endurance trial. All endurance trials required participants to squeeze the handgrip dynamometer at 50% MVC (i.e., average of the two highest baseline MVCs for that study visit) until exhaustion. The target force line (50% MVC) was shown as a static white dotted target line on a computer screen positioned in front of them. Participants were provided with visual feedback on the screen in the form of a force tracing line (i.e., a real-time graphed line that indicated how much force was being generated). Participants were instructed to sustain a handgrip squeeze for as long as possible that kept their active force tracing line as close to the static white dotted target line (50% MVC) as possible. The threshold for the start of an endurance trial was determined when the force exceeded at least 45% MVC for 1 second, while the end was determined to be when the force dropped below 45% MVC for at least 1 second. The time between these start and end thresholds were taken to be the handgrip endurance time and was determined programmatically via custom LabVIEW software.

#### Experimental Sessions (Visits 2–5)

During the experimental visits, participants were instrumented with sEMG and a quiet noise trial was collected for both the sEMG and handgrip dynamometer. Participants then completed three baseline MVCs (as explained previously) followed by the perceived mental fatigue (PMF), perceived physical fatigue (PPF), and the feeling scale (FS). Participants then completed their respective experimental manipulation and provided ratings of PMF, PPF, and FS at 3-minute intervals throughout the experimental manipulation. The study consisted of four experimental manipulations (conditions) that were each conducted for 15 minutes:

##### Cognitive Exertion Condition

Consistent with previous research (Mehta & Parasuraman, 2014), a mental arithmetic task was used as the cognitive exertion manipulation. Participants verbally counted aloud backwards by sevens from a random number between 400 and 900 for 15 minutes. The starting number randomly changed every 3 minutes during the experimental manipulation. This was done to ensure participants did not pick up patterns when subtracting and to keep the task as cognitively demanding as possible. The participant’s accuracy was not recorded; however, participants were monitored to ensure they adhered to the task as closely as possible.

##### Physical Exertion Condition

Participants performed an intermittent isometric handgrip protocol which required them to squeeze a handgrip dynamometer, in the same handgrip MVC posture, intermittently at 15.3% of their baseline MVC. This particular exertion level was chosen as this is the maximum acceptable effort recommended for a repetitive intermittent work task with a 50% duty cycle (i.e., 15 seconds of exertion every 30 seconds of total elapsed time) (Potvin, 2012).

##### Concurrent Exertion Condition

In the concurrent fatigue condition, participants engaged in the tasks described above in the physical exertion and cognitive exertion conditions. That is, participants performed the intermittent isometric handgrip task (15 seconds exertion, followed by 15 seconds of rest) as well as the arithmetic test for 15 minutes.

##### Control Condition

Participants watched a 15-minute documentary film on YouTube titled “Our Planet | Fresh Water” and completed a 15-second exertion at 15.3% MVC at the start, middle (7.5 minutes), and end of the experimental manipulation. This documentary video manipulation has been used in previous research as a control condition (Brown & Bray, 2017).

Following the experimental manipulations, participants performed a postmanipulation MVC and then had 3 minutes to rest while they completed the task self-efficacy (TSE) scale and intrinsic motivation inventory (IMI) scales (described in following section). Participants then completed a handgrip endurance trial followed by ratings of RPE, PMF, PPF, and FS. Once these ratings were completed, the experimenter then assisted the participants in removing the sEMG and the experimental session was concluded for that day.

### Data Analysis

#### Primary Outcome Measures

In addition to grip strength and endurance time (described earlier), several other neuromechanical signals were recorded during the experimental manipulations and subsequent endurance tasks.

##### Muscle Activity

sEMG signals were smoothed using a 0.250 s RMS window. For baseline MVC trials, the RMS signals were additionally smoothed with a half-second moving average before obtaining the peak amplitude from each channel for sEMG normalization (Fischer et al., 2010). Mean RMS amplitudes were taken from a 2.5-second window at the start, 25%, 50%, 75%, and end of the endurance trials and normalized to the peak amplitude during the baseline MVC trials or maximum voluntary excitation (MVE) from that day’s session, creating the normalized muscle activity measure (%MVE).

##### EMG Mean Power Frequency (MnPF)

MnPF was determined using Hanning window and fast fourier transformations computed in 0.5-second window lengths with 50% overlap. From these smoothed MnPF signals, average MnPF within a 2.5-second window length during the beginning and end, as well as the 25%, 50%, and 75%, time points of the endurance trials were computed. MnPF at 25%, 50%, 75%, and 100% time points was normalized to baseline MnPF, creating a normalized MnPF measure (%MnPF), which allows for interpretation of the MnPF decline from baseline for each experimental session.

##### Handgrip Force Fluctuation and Force Tracing Error

Force fluctuations (i.e., force variability) were extracted from the handgrip force data collected during the experimental manipulations and the endurance trials. An increase in force fluctuation is linked to neuromuscular fatigue and reduced task performance (Enoka et al., 2003; Mehta & Agnew, 2012). Force fluctuation was calculated by computing the coefficient of variation between a defined 2.5-second window at the start, 25%, 50%, 75%, and end of the endurance trials. Similarly, force tracing performance was tracked by calculating the absolute error, constant error, and root-mean-square error between the actual force and target force during the same 2.5 second windows throughout the endurance trials.

#### Secondary Outcome Measures

##### Ratings of Perceived Exertion

Participants subjectively rated their perceived exertion (RPE) following each experimental endurance trial using Borg’s CR-10 (Borg, 1998) scale to determine the extent to which they exerted their maximum physical effort on each trial. Participants were asked to rate their perceived physical exertion from 0 (nothing at all) to 10 (absolute maximum).

##### Perceived Mental Fatigue and Perceived Physical Fatigue

Participants rated their perceived mental fatigue (PMF) and perceived physical fatigue (PPF) using two separate visual analogue scales (VAS; Wewers & Lowe, 1990). To complete each measure, participants were asked to mark the point on the line that represents their current state of mental or physical fatigue. Participants marked an “X” along a 100 mm line between 0 (no fatigue) on the left side to 100 (maximum fatigue) on the right side to indicate their perception of their current state of mental or physical fatigue. The scores were calculated by measuring the distance in millimeters that the “X” was placed from the left side of the scale. The PMF scale preceded the PPF scale at each administration. The scales were assessed during the experimental visits following the three baseline MVCs, at five time points during the experimental manipulations (i.e., at 3 min, 6 min, 9 min, 12 min, and 15 min) and following each endurance trial.

##### Affect

The Feeling Scale (FS) is a 11-point bipolar scale developed by Hardy and Rejeski (1989) and is used to measure the participant’s overall affective state, ranging from very good (+5) to very bad (−5) or neutral (0) in the middle. During the experimental visits, participants were asked to report how they were currently feeling after the three baseline MVCs, at five time points during the experimental manipulations (i.e., at 3 min, 6 min, 9 min, 12 min, and 15 min), and following each endurance trial.

##### Motivation

The effort and importance subscale from the Intrinsic Motivation Inventory (IMI; Ryan, 1982) was used to assess participants’ motivation to complete the handgrip endurance trials during the experimental visits. The effort and importance subscale is a 5-item 7-point Likert scale ranging from 1 (not true) to 7 (very true). Each item was prefaced with the following stem: *“For the endurance handgrip task I’m about to do”* and an example item is: *“I am going to put a lot of effort into this task.”* Internal consistency for each measurement was good (Cronbach’s α’s < 0.82).

##### Task Self-Efficacy

Based on recommendations by Bandura (1997, 2006), task self-efficacy (TSE) for performing the experimental endurance trials was assessed using one-item. Specifically, the task self-efficacy scale asked participants *“How confident are you in your ability to perform the handgrip endurance task when compared to the training day (i.e., hold it for a longer period of time).”* Participants rated their confidence using an 11-point, 0 (Not Confident) to 10 (Totally Confident), scale. The participants were reminded that the “training day” referred to their first visit to the lab (i.e., the familiarization visit) and completed the task self-efficacy scale prior to the endurance trial during each experimental visit. A similar scale has been used in previous research when assessing the effects of cognitive exertion on handgrip endurance performance (Graham & Bray, 2015).

### Statistical Analysis

Data visualizations and statistical analyses were performed using R (version 4.0.3 - R Foundation for Statistical Computing, Vienna, Austria) and RStudio (RStudio Inc., Boston, MA, USA) statistical software. To assess the effects of the experimental conditions on the various primary outcome measures, mixed-effects models were fitted using restricted maximum likelihood. For the handgrip endurance time and handgrip strength difference measures, the experimental condition was the fixed effect of interest, with participant ID included as a random effect. For all other primary outcome measures, time window was added as a fixed effect. If homogeneity of the residuals and/or normality of the residuals was violated, a log-transformation was performed on the dependent variable to satisfy model assumptions. Repeated measures ANOVA tables were generated with *p*-values for *F*-tests using Satterthwaite’s method to inform post hoc comparisons (Satterthwaite, 1946). For the handgrip MVCs, estimated marginal means and their 95% confidence intervals were calculated with the null hypothesis that the mean pre–post difference in MVC strength was zero for each experimental condition. Furthermore, any significant main effects of the mixed effects models were further explored using Tukey corrected pairwise comparisons of the estimated marginal means of the model. All pairwise comparisons were two-sided and considered significant if Tukey adjusted *p* < .05. Denominator degrees of freedom were calculated using the Kenward-Roger Degrees of Freedom Approximation (Kenward & Roger, 1997). If possible, pairwise comparisons were performed on the transformed primary outcome measures, then back-transformed to improve interpretability of the findings.

Univariate repeated measures ANOVAs were computed to assess differences in means for the secondary outcome measures. Significant main effects were decomposed and evaluated using Tukey’s (LSD) post hoc comparisons of the estimated marginal means and their 95% confidence intervals. All pairwise comparisons were two-sided and considered significant if Tukey adjusted *p* < .05. Effects sizes for the repeated measures ANOVAs are reported as partial eta squared ( $\eta_{P}^{2}$ ) and the values for small, medium, and large are 0.01, 0.06, and 0.14, respectively. Effect sizes for the post hoc comparisons are reported as Cohen’s *d* (Cohen, 1992) and the values for small, medium, and large are 0.20, 0.50, and 0.80.

## Results

### Primary Outcome Measures

#### Handgrip MVC Strength

**Figure 3.**
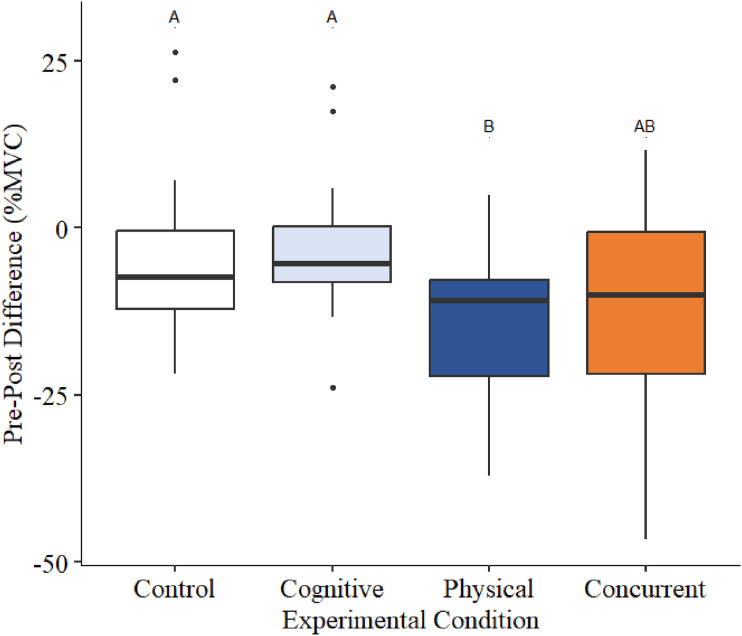
Pre–post differences for grip strength (%MVC) for the four experimental conditions. Differences between each condition are statistically significant if they do not share a letter (*p* < 0.05). Outliers are classified as an observation that is outside the interquartile range multiplies by 1.5.

Estimated marginal means for percentage differences in pre-to-post MVC strength were not significantly different from zero within the control (−4.85 95%CI [−9.86, 0.169], *t* (83.1) = −1.922, *p* = .058) and cognitive conditions (−3.47 [−8.49, 1.54], t (83.1) = −1.38, *p* = .172). However, the mean percentage differences for the physical (−14.38 [−19.4, −9.36], *t* (83.1) = −5.70, SEM = 2.52, *p* = <.0001) and concurrent condition (−11.63 [−16.64, −6.61], *t* (83.1) = −4.61, SEM = 2.52, *p* = <.0001) were significantly lower than zero. There was a main effect for experimental condition (*F* (3,72) = 5.63, *p* = 0.0016; Figure 3, Table 1). The physical condition resulted in a significantly larger decline in MVC strength between the pre-to-post MVC when compared to the cognitive condition (*t* (72) = 2.641, SEM = 3.36, *p* = .0046). Additionally, the pre-to-post MVC difference in the physical condition was 9.5% lower than the control condition, as a percentage of MVC (*t* (72) = 3.042, SEM = 3.13, *p* = .0169).

**Table 1.** Descriptive Statistics for Handgrip MVC.

Primary Outcome Measures	Condition			
	Control	Cognitive	Physical	Concurrent
	*M* (*SD*)	*M* (*SD*)	*M* (*SD*)	*M* (*SD*)
Baseline MVC (*N*)	313.26 (102.04)	305.49 (112.55)	299.38 (99.74)	308.64 (106.63)
MVC post EM (*N*)	302.61 (117.22)	293.73 (106.89)	259.76 (105.64)	274.17 (112.92)

*Note. M* = mean, *SD* = standard deviation, *N* = newtons, MVC = maximum voluntary contraction.

#### Handgrip Endurance Time

**Figure 4.**
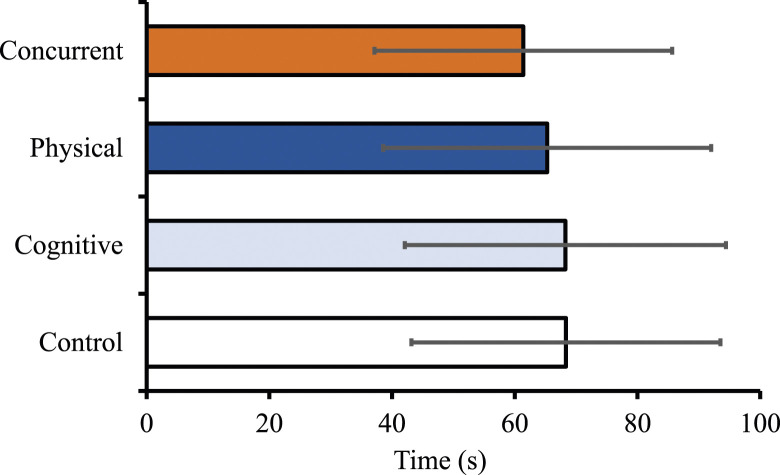
Grip endurance times following the four experimental manipulations. Error bars indicate standard deviation.

The main effect of the experimental condition was not significant for endurance time (*F* (3,72) = 1.67, MSE = 268.74, *p* = .182) (Figure 4).

#### Handgrip Force Fluctuation

**Figure 5.**
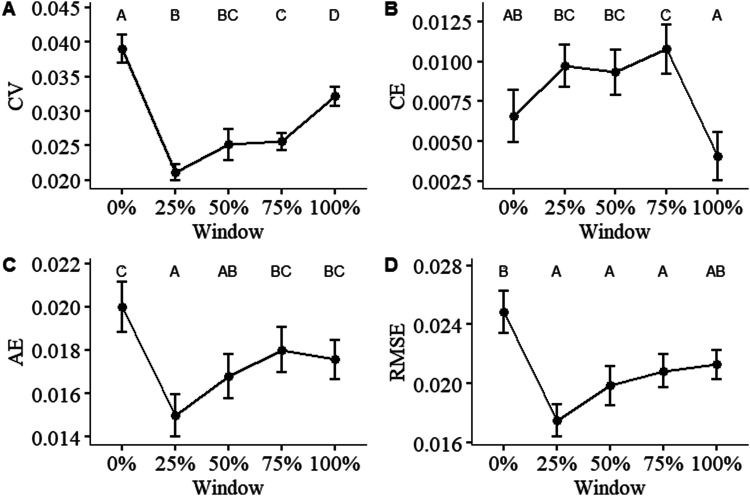
The figure is separated by measurement method: (a) force fluctuation, shown as coefficient of variation (CV); (b) constant error (CE); (c) average error (AE); (d) root-mean-square error (RMSE). Differences in means between each time point are statistically significant if they do not share a letter (*p* < .05). Error bars indicate standard error (*n* = 100).

**Figure 6.**
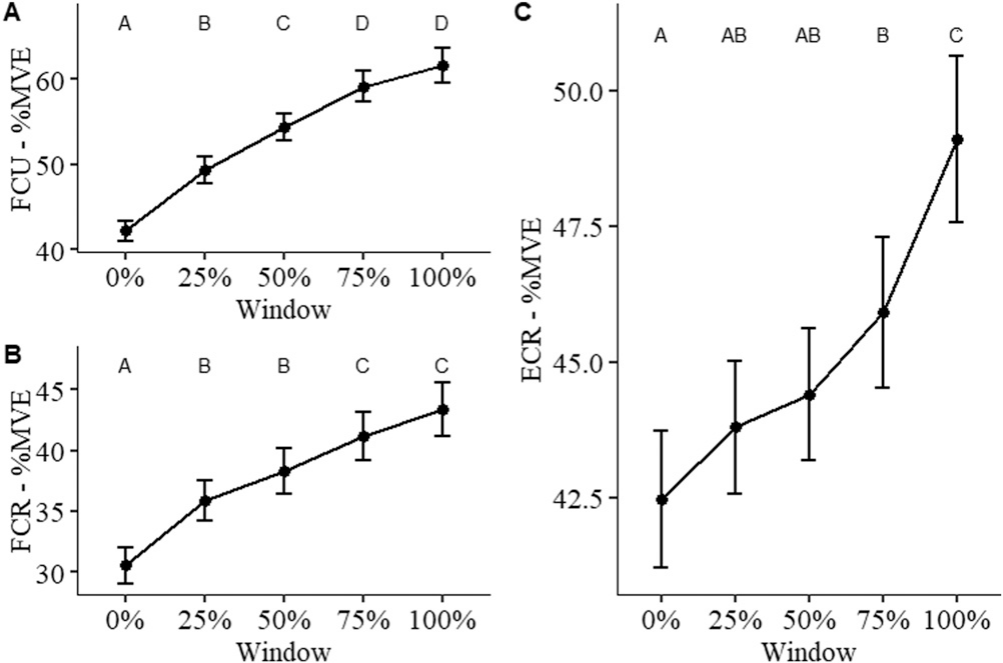
sEMG amplitude as a percentage of maximum voluntary exertion (%MVE) over the endurance trial completed. Muscles measured include (a) flexor carpi ulnaris (FCU), (b) flexor carpi radialis (FCR), and (c) extensor carpi radialis (FCR). Differences in means between each timepoint are statistically significant if they do not share a letter (*p* < .05). Error bars indicate standard error (*n* = 100).

For all force fluctuation and tracing error measures, the main effect of condition and its interaction with time were not significant. Nonetheless, the main effect of time was significant for coefficient of variation (CV; Figure 5(a) - *F* (4,456) = 43.096, MSE = 7.443, *p* < .001), constant error (CE; Figure 5(b)–*F* (4, 384) = 6.745, MSE = 1.78, *p* < .001), absolute error (AE; Figure 5(c) – *F* (4,456) = 8.8129, MSE = 1.77724, *p* < .001), and root-mean-square error (RMSE; Figure 5(d); Figure 6(d) – *F* (4,456) = 6.919, MSE = 0.0007, *p* < .001).

#### sEMG %MVE

For %MVE of all muscles, the main effect of condition and its interaction with time were not significant. However, the main effect of time was significant for %MVE of the FCU (Figure 6(a) – *F* (4, 381.08) = 56.96, MSE = 2.197, *p* < .001), FCR (Figure 6(b) – *F* (4, 381.96) = 57.99, MSE = 1.79, *p* < .001), and ECR (Figure 6(c) – *F* (4, 379.06) = 13.05, MSE = 0.2912, *p* < .001).

#### sEMG %MnPF

**Figure 7.**
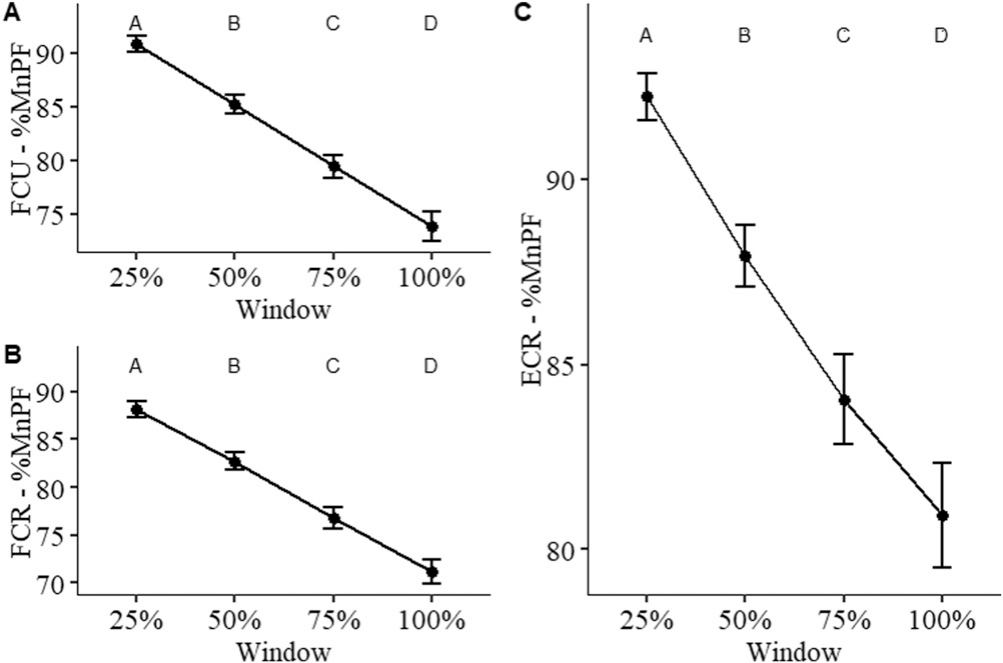
Mean power frequency of the sEMG normalized to the baseline mean power frequency (%MnPF) during the endurance trials. Muscles measured include: (a) flexor carpi ulnaris (FCU), (b) flexor carpi radialis (FCR), and (c) extensor carpi radialis (FCR). Differences in means between each time point are statistically significant if they do not share a letter (*p* < .05). Error bars indicate standard error (*n* = 100).

For %MnPF of all muscles, the main effect of condition and its interaction with time were not significant. Although, the main effect of time was significant for %MnPF of the FCR (Figure 7(a) – *F* (4, 286.396) = 142.42, MSE = 1.825, *p* < .001), FCU (Figure 7(b) – *F* (3, 285) = 179.65, MSE = 1.93, *p* < .001), and ECR (Figure 7(c) – *F* (3, 274.34) = 49.102, MSE = 0.88, *p* < .001). Further statistical summaries can be found in the supplemental material.

### Secondary Outcome Variables and Manipulation Checks

Descriptive statistics for the secondary outcome measures are shown, by condition, in Table 2.

**Table 2.** Descriptive Statistics for Secondary Outcome Measures.

Measures	Condition			
	Control	Cognitive	Physical	Concurrent
	*M* (*SD*)	*M* (*SD*)	*M* (*SD*)	*M* (*SD*)
RPE postendurance trial	6.32 (2.83)	6.18 (2.80)	6.46 (2.31)	6.54 (2.56)
PMF baseline	12.76 (14.54)	16.82 (16.90)	17.88 (21.73)	15.80 (19.52)
PMF postmanipulation	15.00 (16.72)	24.78 (24.78)	22.89 (21.89)	41.66 (21.67)
PMF postendurance trial	15.00 (16.72)	39.70 (24.78)	22.88 (21.89)	41.66 (21.67)
PPF baseline	16.10 (18.10)	16.88 (15.43)	15.92 (19.50)	19.04 (18.91)
PPF postmanipulation	34.56 (23.99)	42.34 (25.54)	52.76 (22.55)	51.68 (24.97)
PPF postendurance trial	35.56 (23.99)	42.34 (25.54)	52.76 (22.55)	51.68 (24.97)
Affect baseline	3.04 (1.77)	2.60 (2.00)	2.68 (2.25)	2.44 (1.96)
Affect postmanipulation	2.84 (1.34)	2.16 (1.97)	2.34 (1.76)	1.68 (1.91)
Affect postendurance trial	2.84 (1.34)	2.16 (1.97)	2.34 (1.76)	1.68 (1.91)
Motivation	6.16 (0.71)	6.01 (0.91)	6.07 (0.80)	6.06 (0.86)
Task self-efficacy	7.64 (1.85)	6.04 (2.56)	6.08 (2.48)	5.44 (2.58)

*Note. M* = mean, *SD* = standard deviation, PMF = perceived mental fatigue, PPF = perceived physical fatigue, RPE = ratings of perceived exertion. The scales for PMF and PPF range from 0 (no perceived fatigue) to a maximum of 100 (maximum perceived fatigue). The RPE scale ranges from 0 (no perceived exertion) to 10 (maximum perceived exertion).

#### Manipulation Checks

PMF, PPF, and affect (via FS) ratings were assessed during the experimental manipulations to assess the extent to which the experimental conditions may have differently impacted participants’ perceptions of mental fatigue, perceptions of physical fatigue, and affect over the course of the experimental manipulation.

##### Perceived Mental Fatigue (PMF), Physical Fatigue (PPF) and Affect (FS)

**Figure 8.**
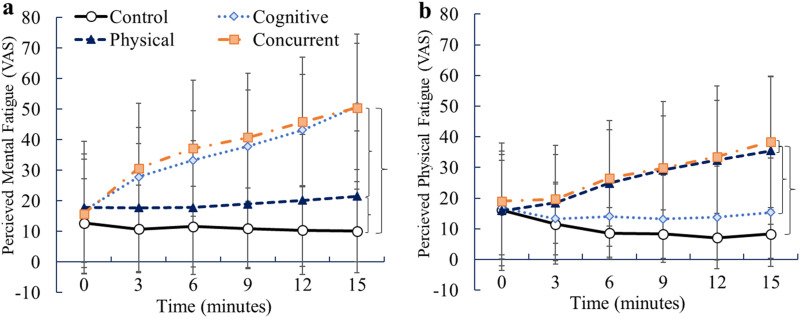
Perceived mental (a) and physical (b) fatigue throughout the experimental manipulation, separated by experimental condition. Error bars indicate standard deviations and brackets “}” indicate significant differences (main effect) between conditions, collapsed across all time points.

There was a significant main effect for PMF scores (*F* (3, 72) = 18.81, *p* < .001, $\eta_{P}^{2}$ = 0.56) and PPF scores (*F* (3, 72) = 14.00. *p* < .001, $\eta_{P}^{2}$ = 0.37). Average PMF scores in the cognitive condition were significantly higher than the control (*p* < .001, *d* = 1.72) and physical (*p* < .001, *d* = 0.88) conditions. PMF scores in the concurrent condition were also significantly higher than the control (*p* < .001, *d* = 1.56) and physical (*p* = .002, *d* = 0.88) conditions. Finally, PMF scores in the physical condition were significantly higher than the control condition (*p* = .03, *d* = 0.46) (Figure 8(a)). Average PPF scores in the physical condition were significantly higher than the control (*p* < .001, *d* = 1.01) and cognitive (*p* = .007, *d* = 0.67) conditions. PPF scores in the concurrent condition were also significantly higher than the control (*p* < .001, *d* = 1.38) and cognitive (*p* < .001, *d* = 0.93) conditions (Figure 8(b)).

##### Affect

There was a significant main effect for feeling scale (FS) scores, *F* (3, 72) = 6.37, *p* = .005, $\eta_{P}^{2}$ = 0.16. As seen in Table 2, average FS scores were significantly higher in the control condition when compared to the cognitive (*p* = .009, *d* = 0.52), physical (*p* = .04, *d* = 0.38), and concurrent (*p* < .001, *d* = 0.66) conditions.

#### Secondary Outcome Measures

The secondary outcome measures represent subjective measures taken prior to and following the endurance trials (bottom of Table 2).

##### Ratings of Perceived Exertion (RPE)

There was no main effect on RPE scores following the endurance trial (*F* = 0.37, *p* = .78, $\eta_{P}^{2}$ = 0.02).

##### Perceived Mental Fatigue (PMF)

There were no baseline differences for PMF scores (*F* = 0.67, *p* = .58, $\eta_{P}^{2}$ = 0.03). However, there was a significant main effect on PMF scores following the endurance trial (*F* = 10.75, *p* < .001, $\eta_{P}^{2}$ = 0.31). PMF scores in the cognitive condition were significantly higher than the control (*p* < .001, *d* = 1.17) and physical (*p* = .02, *d* = 0.72) conditions. PMF scores in the concurrent condition were also significantly higher than the control (*p* < .001, *d* = 1.38) and physical (*p* = .002, *d* = 0.86) conditions.

##### Perceived Physical Fatigue (PPF)

There were no baseline differences for PPF scores (*F* = 0.40, *p* = .75, $\eta_{P}^{2}$ = 0.02). However, there was a significant main effect on PPF scores following the endurance trial (*F* = 4.90. *p* = .004, $\eta_{P}^{2}$ = 0.17). PPF scores in the control condition were significantly lower than the physical (*p* = .001, *d* = 0.74) and concurrent (*p* = .003, *d* = 0.66) conditions.

##### Affect, Motivation, and Task Self-Efficacy

There were no baseline differences for FS scores (*F* = 1.23, *p* = 0.31, $\eta_{P}^{2}$ = 0.05). However, there was a significant main effect on FS scores following the endurance trial (*F* = 4.71, *p* = .005, $\eta_{P}^{2}$ = 0.16). FS scores in the control condition were significantly higher than the cognitive (*p* = .03, *d* = 0.40) and concurrent (*p* < .001, *d* = 0.70) conditions. There was no main effect for the effort and importance subscale scores from the Intrinsic Motivation Inventory (IMI) assessed prior to the endurance trial (*F* = 0.50, *p* = .69, $\eta_{P}^{2}$ = 0.02). There was a significant main effect for the TSE scores assessed prior to the endurance trial (*F* = 8.83, *p* < .001, $\eta_{P}^{2}$ = 0.27). TSE scores in the control condition were significantly higher than the cognitive (*p* = .002, *d* = 0.72), physical (*p* = .002, *d* = 0.71), and concurrent (*p* < .001, *d* = 0.98) conditions.

## Discussion

This study investigated the independent and interactive carryover effects of fatiguing cognitive and physical exertion on various aspects of physical performance (i.e., handgrip strength, endurance, muscle activity, and force tracing). Based on previous research (Alder et al., 2021), we hypothesized that the concurrent exertion condition (cognitive and physical exposures combined) would have the greatest negative carryover effects on physical task performance, followed by the cognitive exertion and physical exertion conditions. Our study found significant differences in grip strength following only the physical condition compared to cognitive and the control condition; though the concurrent condition was shown to be significantly different from baseline strength. Similar to the findings of Mehta and colleagues (2012, 2014), concurrently completing a physical and cognitively demanding task was no different than the physical task alone. Both the control and cognitive conditions had no significant changes from baseline grip strength. For the endurance task following the experimental manipulations, the concurrent condition had an 11.8% reduction in performance from baseline, and the physical condition had a 9.2% lower mean performance, but these differences were not significant. In fact, the control condition also had a 5% lower endurance time, which likely reflects the high variability often observed with endurance trials (Reuter et al., 2011; Watterworth et al., 2022).

In addition to strength and endurance performance, this study also evaluated the effects of these conditions on both force tracing and EMG during the subsequent endurance task. All EMG and force metrics showed significant manifestation of fatigue, as would be expected during a sustained isometric contraction at 50% MVC, but again, no differences emerged between the different exposure conditions. For force fluctuation and measures of tracing performance (i.e. tracing RMSE, CE, and AE), there were significant improvements in performance between the start of the endurance trial to the 25%-time window, but then a progressive increase in CV and tracing error as fatigue accumulated through the remainder of the endurance task. The initial improvement is likely attributable to the stabilization to the 50% MVC force level upon initially beginning the task (i.e., ramping up force and settling the grip force around a steady-state level). The EMG signals also had a progressive increase in amplitude, and decrease in mean power frequency, as would be expected during a fatiguing sustained isometric task (Cifrek et al., 2009). These differences were consistent across the 4 conditions, with no significant differences in fatigue manifestation.

The diminutive responses observed for condition on endurance time and task performance point to a further examination of the manipulations themselves. How effective were they in inducing physical and mental fatigue? When combined, perceptions of mental and physical fatigue were highest in the concurrent condition following the experimental manipulation, supporting the effectiveness of the manipulation. Similarly, perceptions of physical fatigue or mental fatigue were higher than the control condition with regards to their respective condition. These findings suggest that the manipulations were effective at inducing theorized changes in perceptions of physical and/or mental fatigue, yet we did not observe subsequent changes in endurance task performance found in previous research. In addition, we also observed that self-efficacy scores to perform the endurance task were significantly higher in the control condition when compared to the other conditions which has been found in previous research (Graham & Bray, 2015; Graham et al., 2017); however, changes in self-efficacy ultimately accounted for (i.e., mediated) subsequent changes in physical task performance in those studies. Despite the effectiveness of the experimental conditions with regards to changes in perceptions of fatigue and self-efficacy, another psychological variable may have been attributed to the nonsignificant effects found for endurance performance. For instance, motivation to perform the endurance trial was high (above 6/7) and consistent across conditions. It is plausible high levels of motivation minimized the effects of different types of exertions on physical task performance whereby participants were willing to exert more effort to perform the endurance trial and/or resist the temptation to quit as muscle fatigue increased.

In addition to the physical task performance and psychological outcomes, this study also evaluated myoelectric and force variability responses during the endurance trials. Consistent with prior literature, sEMG amplitude and force variability measures increased over the duration of the isometric endurance trial, (Häkkinen & Komi, 1983; Missenard et al., 2008), while mean sEMG spectral power decreased (Häkkinen & Komi, 1983). However, these trends were consistent across conditions. When considered in concert with the handgrip MVC and endurance time findings, it is possible that the physical exposure may not have been potent enough. A relative handgrip contraction level of 15.3% MVC was utilized for the physical and concurrent protocols, as this is the estimated maximum acceptable effort for a repetitive upper limb task conducted with a 50% duty cycle (similar to an “on-off” ratio) (Potvin, 2012). This contraction level represents the ergonomics limit, above which excessive fatigue is expected to accumulate. As such, we selected this contraction level as a balance point to ensure a moderate effort level that would not lead to exhaustion during the 15-minute trials, and to be an exposure representative of repetitive occupational tasks. Future research should repeat this study at higher relative cognitive and physical workloads to determine if the concurrent task would indeed be worse than physical or cognitive fatigue alone, when at more extreme (i.e., fatiguing) exposure levels.

The results from this study need to be interpreted in light of several limitations. Despite previous research commonly using endurance time as a dependent measure following cognitive and physical exertion, results using endurance time need to be interpreted with caution due to its inherently poor test-retest reliability (Reuter et al., 2011; Watterworth et al., 2022). Reuter and colleagues (2011) instead suggest that pre- and postfatigue MVCs be utilized as the primary outcome measure of interest in handgrip studies, due to its excellent test-retest reliability in healthy individuals and well-established validity (Boissy et al., 1999; Massy-Westropp et al., 2011; Reuter et al., 2011). The differences in reliability between these two measures may explain the lack of notable findings for the endurance time measure, but significant findings for pre to post MVC differences in the present study. Although it is noteworthy that we did not find changes in endurance time following the cognitive condition, which has been consistently observed in previous research (Brown et al., 2020), we utilized a cognitive task (i.e., mental arithmetic) that was chosen based on the successful execution of the concurrent manipulation. Pilot testing revealed that the Stroop task, commonly used in previous research following cognitive exertion (Brown et al., 2020), was too demanding/distracting whereby participants were unable to perform the intermittent handgrip task as required. Mental arithmetic was chosen given it has been used in previous studies examining concurrent exertion (Mehta & Parasuraman, 2014); however, it is plausible it is not as mentally fatiguing as the Stroop task for which previous research has shown negative carryover effects from the Stroop task on neuromuscular and endurance handgrip outcomes (Bray et al., 2008; Brown & Bray, 2017). Sex-related differences in physiology and anatomy, as highlighted in studies such as the one by Hunter (2014), play a significant role in neuromuscular performance and fatigability. However, due to the relatively small sample size (12 females, 13 males), robust comparisons between the sexes were not feasible within the scope of this study. Future studies with larger sample sizes are warranted to explore these relationships more comprehensively.

It is also very likely that the relative lack of effects seen in this study could be partially explained by the intensity and duration of both the cognitive and physical exertions. The concurrent nature of our protocol required use of an arithmetic task, rather than an incongruent Stroop task, as visual attention was required to match the force level during the handgrip exertions. Although a handful of previous studies have found negative carryover effects following an arithmetic manipulation on task performance, many more studies have utilized a Stroop task and found consistent negative effects on handgrip performance (see Table 1 from Brown et al., 2020). It is possible the Stroop task requires higher levels of cognitive exertion compared to an arithmetic task. Indeed, previous research found that mental fatigue scores following 10 minutes of a Stroop task were on average 60/100 or higher (Brown et al., 2017) whereas mental fatigue scores in the present study were on average 36/100 following mental arithmetic. Future research is encouraged to replicate the present study and utilize a longer mental arithmetic manipulation or develop a concurrent exertion manipulation that includes Stroop task performance that would lead to higher scores of mental fatigue. However, despite the moderate exposures, strength was affected after only 15 minutes for the cognitive and physical conditions. One can extrapolate how these effects may continue to manifest themselves over an entire workday. A future study that either increases the exposure levels (i.e., utilizing a modified Stroop task and increasing the relative force level), or conducting the manipulation over a longer time period, may very well establish further negative performance decrements during concurrent physical and cognitive exposures.

## Conclusion

In conclusion, this study examined the effect of physical, mental, and concurrent fatigue exposures relative to a control condition, on several performance and neuromechanics-based response variables. Both the concurrent and physical fatigue conditions resulted in significant reductions in handgrip strength compared to zero. However, the concurrent condition was no worse than the physical fatigue condition at the relatively moderate workloads examined in this study. Future research should further examine these manipulations over longer durations, or at higher relative physical and cognitive exposure levels, to further elucidate the effects of concurrent exertion on physical performance.

## Key Points

• This study investigated the independent and interactive effects of cognitive and physical demands on aspects of physical performance.
• Findings demonstrate that tasks requiring concurrent cognitive and physical demands can have negative carryover effects on task self-efficacy and muscular strength.
• Occupational tasks that elicit high levels of simultaneous cognitive and physical effort should be avoided when subsequent tasks require muscular strength of the upper extremity.

## Supplemental Material

Supplemental Material - Examining the Independent and Interactive Carryover Effects of Cognitive and Physical Exertions on Physical Performance Supplemental Material for Examining the Independent and Interactive Carryover Effects of Cognitive and Physical Exertions on Physical Performance by Rahul K. Pabla, Jeffrey D. Graham, Michael W. B. Watterworth and Nicholas J. La Delfa in Human Factors
