# Supplementary material for: Examining the Independent and Interactive Carryover Effects of Cognitive and Physical Exertions on Physical Performance
Source: Hum Factors. 2024 Oct 21;67(6):560–77. doi: 10.1177/00187208241293720 (PMC12049582; doi:10.1177/00187208241293720)
Supplement: Supplemental Material - Examining the Independent and Interactive Carryover Effects of Cognitive and Physical Exertions on Physical Performance [file sj-pdf-1-hfs-10.1177_00187208241293720.pdf]

Submitted to Human Factors

Appendix A – Pairwise Comparisons by Time Point for Force Fluctuations, %MVE, and %MnPF.

Handgrip Force Fluctuation

The CV at the 0% time point was significantly greater than at 25% ($t(456) = 8.481$, SEM = 0.002, $p < .001$), 50% ($t(456) = 6.633$, SEM = 0.002, $p < .001$), 75% ($t(456) = 6.349$, SEM = 0.002, $p < .001$), and 100% ($t(456) = 3.239$, SEM = 0.002, $p = .0112$). Additionally, the CV at the 100% time point was significantly greater than at 25% ($t(456) = 5.242$, SEM = 0.002, $p < .001$), 50% ($t(456) = 3.394$, SEM = 0.002, $p < .001$), and 75% ($t(456) = 3.11$, SEM = 0.002, $p < .001$).

The CE at the 0% time point was significantly greater than at 75%. The CE at the 100% time point was significantly less than the CE at 25% ($t(384) = 3.813$, SEM = 0.00149, $p = .0015$), 50% ($t(456) = 3.538$, SEM = 0.001489, $p = .0041$), and 75% time points ($t(456) = 4.498$, SEM = 0.001489, $p < .001$).

The AE at the 0% time point was significantly greater than at 25% ($t(456) = 5.56$, SEM = 0.0904, $p < .001$), and 50% ($t(456) = 3.341$, SEM = 0.0506, $p = .008$). The AE at the 25% time point was significantly greater than at 75% ($t(456) = 3.565$, SEM = 0.0506, $p = .0037$), and 100% ($t(456) = 4.086$, SEM = 0.0490, $p < .001$).

The RMSE at the 0% time point was significantly greater than at 25% ($t(456) = 5.131$, SEM = 0.00143, $p < .001$), 50% ($t(381) = 3.486$, SEM = 0.0193, $p = .0049$), and 75% ($t(381) = 2.789$, SEM = 0.0193, $p = .0436$).

sEMG %MVE

FCU amplitude at 0% was significantly lower than at the 25% ($t(381) = -5.28$, SEM = 0.0241, $p < .001$), 50% ($t(381) = -8.935$, SEM = 0.0217, $p < .001$), 75% ($t(381) = -11.86$, SEM =

For Review Puposes Only

0.02, $p < .001$), and 100% time points ($t(381) = -13.17$, SEM = 0.0193, $p < .001$). FCU amplitude at 25% was also significantly lower than at the 50% ($t(381) = -3.648$, SEM = 0.0252, $p = .0028$), 75% ($t(381) = -6.574$, SEM = 0.0232, $p < .001$), and 100% time points ($t(381) = -7.88$, SEM = 0.0224, $p < .001$). FCU amplitude at 50% was significantly lower than at the 75% ($t(381) = -2.93$, SEM = 0.0257, $p = .0298$), and 100% time points ($t(381) = -4.23$, SEM = 0.0248, $p < .001$).

%MVE of the FCR at 0% was significantly lower than at 25% ($t(382) = -6.59$, SEM = 0.0211, $p < .001$), 50% ($t(382) = -9.25$, SEM = 0.0198, $p < .001$), 75% ($t(382) = -12.18$, SEM = 0.0184, $p < .001$), and 100% ($t(382) = -13.599$, SEM = 0.0177, $p < .001$). %MVE of the FCR at the 25% time point was also significantly lower than the 75% ($t(382) = -5.609$, SEM = 0.0217, $p < .001$), and 100% time points ($t(382) = -7.01$, SEM = 0.0209, $p < .001$). A similar finding occurred for the 50% time point, where %MVE of the FCR was significantly lower than at the 75% ($t(382) = -2.92$, SEM = 0.0233, $p =.03$), and 100% time points ($t(382) = -4.31$, SEM = 0.0224, $p < .001$).

%MVE of the ECR at 0% was significantly lower than at 75% ($t(379) = -3.76$, SEM = 0.0196, $p = .0018$), and 100% ($t(379) = -6.812$, SEM = 0.0184, $p < .01$). Oppositely, %MVE of the ECR at the 100% time point was significantly higher than at the 25% ($t(379) = -5.123$, SEM = 0.0190, $p < .001$), 50% ($t(379) = -4.33$, SEM = 0.0194, $p < .001$, and 75% time points ($t(379) = -3.05$, SEM = 0.0199, $p = .0206$).

sEMG %MnPF

For %MnPF of the FCR, all time points were significantly different from each other. Specifically, %MnPF of the FCR at the 25% time point was significantly lower than at 50% ($t(287) = -6.46$, SEM = 0.016, $p < .001$), 75% ($t(287) = -13.73$, SEM = 0.016, $p < .001$), and

100% ($t(287) = -20.57$, SEM = 0.016, $p < .001$). %MnPF of the FCR at the 50% time point was significantly lower than at 75% ($t(287) = -7.23$, SEM = 0.016, $p < .001$), and 100% ($t(287) = -14.05$, SEM = 0.016, $p < .001$). FCR %MnPF at the 75% time point was significantly lower than at 100% ($t(287) = -6.84$, SEM = 0.016, $p < .001$).

. For %MnPF of the FCU, all time points were significantly different from each other. Specifically, %MnPF of the FCU at the 25% time point was significantly lower than at 50% ($t(285) = -8.67$, SEM = 0.015, $p < .001$), 75% ($t(285) = -15.97$, SEM = 0.015, $p < .001$), and 100% ($t(285) = -21.96$, SEM = 0.015, $p < .001$). %MnPF of the FCU at the 50% time point was significantly lower than at 75% ($t(285) = -7.299$, SEM = 0.015, $p < .001$), and 100% ($t(285) = -13.29$, SEM = 0.015, $p < .001$). %MnPF of the FCU at the 75% time point was significantly lower than at 100% ($t(285) = -5.99$, SEM = 0.015, $p < .001$).

. For %MnPF of the ECR, all time points were significantly different from each other. Specifically, %MnPF of the ECR at the 25% time point was significantly lower than at 50% ($t(284) = -5.16$, SEM = 0.019, $p < .001$), 75% ($t(284) = -8.75$, SEM = 0.019, $p < .001$), and 100% ($t(284) = -11.47$, SEM = 0.019, $p < .001$). %MnPF of the ECR at the 50% time point was significantly lower than at 75% ($t(284) = -3.56$, SEM = 0.019, $p = .0025$), and 100% ($t(284) = -6.28$, SEM = 0.019, $p < .001$). Additionally, %MnPF of the ECR at the 75% time point was significantly lower than at 100% ($t(284) = -2.726$, SEM = 0.019, $p = .034$).

Descriptive statistics for the primary outcome measures are shown, by condition, in Table A.1.

Table A.1: Descriptive Statistics for Primary Outcome Measures.

Primary Outcome Measures	Condition			
	Control *M* (*SD*)	Cognitive *M* (*SD*)	Physical *M* (*SD*)	Concurrent *M* (*SD*)
**Force and Endurance Measures**				
Baseline MVC (N)	313.26 (102.04)	305.49 (112.55)	299.38 (99.74)	308.64 (106.63)
MVC post EM (N)	302.61 (117.22)	293.73 (106.89)	259.76 (105.64)	274.17 (112.92)
Pre-to-post EM MVC difference (%MVC)	-4.85 (12.05)	-3.47 (9.17)	-14.38 (12.09)	-11.63 (16.14)
Endurance time (seconds)	68.35 (25.18)	68.25 (27.17)	65.29 (26.72)	61.39 (24.27)
**Force Variability at 100% Window**				
Coefficient of variation	0.03 (0.01)	0.03 (0.01)	0.03 (0.02)	0.03 (0.01)
Constant error	0.004 (0.01)	0.006 (0.02)	0.002 (0.02)	0.005 (0.02)
Absolute error	0.02 (0.01)	0.02 (0.01)	0.02 (0.01)	0.02 (0.01)
Root-mean-square error	0.02 (0.01)	0.02 (0.01)	0.02 (0.01)	0.02 (0.01)
**%MVE at 100% Window**				
Flexor carpi radialis	47.51 (20.88)	43.23 (23.51)	41.02 (22.28)	41.68 (23.94)
Extensor carpi radialis	50.63 (16.22)	48.59 (11.87)	49.15 (18.81)	48.03 (14.36)
Flexor carpi ulnaris	61.18 (20.63)	63.07 (22.16)	59.71 (18.23)	62.31 (19.37)
**%MnPF at 100% Window**				

Flexor carpi radialis	69.59 (12.98)	69.85 (11.53)	70.72 (12.39)	74.47 (11.75)
Extensor carpi radialis	75.33 (15.08)	81.85 (17.16)	84.32 (11.58)	82.30 (10.64)
Flexor carpi ulnaris	72.33 (15.32)	71.02 (12.74)	74.83 (13.13)	77.28 (11.65)

Note: *M* = mean, *SD* = standard deviation, N = newtons, % = percent, MVC = maximum voluntary contraction, MVE = maximum voluntary excitation, MnPF = mean power frequency.
